# Socioeconomic disparities: a more important risk factor for advanced-stage oral cancer in Florida than smoking?

**DOI:** 10.1007/s10552-025-01992-7

**Published:** 2025-03-29

**Authors:** Garrett Forman, Uche C. Ezeh, Isabella Buitron, Sophia Peifer, Liana Shtern, Tonya Aaron, Abdurrahman Al-Awady, Isildinha M. Reis, Erin R. Kaye, Elizabeth Nicolli, David Arnold, Francisco Civantos, Ming Lee, Elizabeth Franzmann

**Affiliations:** 1 Department of Otolaryngology, University of Miami Miller School of Medicine, 1600 NW 10 Avenue, Miami, FL 33136 USA; 2 New York University, New York, NY 10012 USA; 3 Albert Einstein College of Medicine, Bronx, NY 10461 USA

**Keywords:** Oral cancer, Racial disparities, Socioeconomic disparities, Insurance, Education, SEER stage

## Abstract

**Purpose:**

To explore the associations between sociodemographic factors with advanced-stage oral cavity cancer (OCC) presentation among Floridians.

**Methods:**

Demographic and cancer data on OCC patients (*n* = 7,826) diagnosed between 2010 and 2017 were retrieved from the Florida Cancer Data System (FCDS). Census tract median income and percentage of population with a bachelor’s degree or higher were used to infer income and education. Pearson’s chi-square tests of independence were used to compare sociodemographic factors between racial/ethnic groups and staging groups. Multinomial logistic regression analyzed predictors of advanced disease. Incidence and percent late-stage diagnosis versus income were mapped using ArcGIS Pro.

**Results:**

Among 5,252 cases analyzed: 5.7% were Black, 82.4% White Non-Hispanic, 61.5% male, 63.3% publicly insured, 6.5% uninsured, 58.7% current or former smokers, and 73.0% urban residents. Black patients were more likely to present with advanced disease, be single/unmarried, uninsured, and less likely to be former smokers. Male sex, Black race, non-married status, no insurance, Medicaid, VA/military insurance, and lower educational status were associated with increased risk of regional vs. early disease in multivariable analysis (MVA) (*p* < 0.05). These factors, in addition to Medicare, were associated with distant disease in MVA. Geospatial mapping revealed higher rates of regional and distant disease presentation in the Tampa Bay and Orlando areas.

**Conclusion:**

Black race, male sex, non-married status, lower education, Medicaid, VA/Military insurance and no insurance were associated with advanced OCC in Florida. Smoking status was not associated with advanced disease presentation after adjusting for sociodemographic variables.

**Supplementary Information:**

The online version contains supplementary material available at 10.1007/s10552-025-01992-7.

## Introduction

The American Cancer Society estimates there will be 36,000 diagnoses of oral cavity cancer (OCC) in the US in 2024, over 90% of which are squamous cell carcinomas [1]. Unfortunately, the society predicts that 7,930 OCC patients will die from their disease within the same time frame. Despite an overall decline in cancer mortality rates, data indicates a concerning rise in OCC incidence and mortality among certain sociodemographic groups and cancer sites [2–4]. A database study by Farhadi et al. demonstrated a significant increase in OCC and lip cancer mortality between 2009 and 2019, despite overall OCC and oropharyngeal combined mortality remaining stable.

Risk factors traditionally associated with increased OCC incidence and poorer outcomes include cigarette, alcohol, and betel nut consumption [5, 6]. Growing attention has been directed at the impact of socioeconomic factors and socioeconomic status (SES) on OCC. Patients of Black race, low income, low education, non-Hispanic ethnicity, unmarried status, public insurance and no insurance often have increased OCC incidence and worse outcomes [7–20]. Associations between residence (urban vs. rural) and OCC are less clear, although studies have linked rural residence to worse outcomes and rising disparities [21–24]. In Florida specifically, a recent study demonstrated increased OCC incidence among patients who are Black, Hispanic, males, married, and current smokers [25]. However, associations with income, education, and advanced stage were not evaluated.

As anticipated, advanced-stage OCC at diagnosis is associated with increased mortality [26, 27]. Screening guidelines are established tools for reducing morbidity and mortality in other types of cancer [28, 29]. While there are no such guidelines for OCC in the US, screening for this cancer has shown promise in international studies [30, 31]. A screening trial in India by Sankaranarayanan et al. demonstrated significantly decreased long-term mortality in screened males with risk factors (i.e., tobacco use). Evaluation of an OCC detection program in Taiwan by Chuang et al. also found significant improvements in survival among participants. Due to the findings of these studies and the high costs of healthcare, greater benefits would likely be seen when targeting such programs at the highest-risk individuals. Although populations facing OCC disparities are often more likely to engage in smoking and other risky behaviors, additional factors relating to demographics and SES may play a role [7, 9, 19, 32]. Contributing factors leading to the increased risk in low SES groups, including unmarried individuals and the uninsured, may include decreased healthcare utilization, dietary differences, and decreased social support [33, 34]. Knowledge of these populations, as well as general SES factors associated with delayed diagnosis, will be critical in the development of screening trials and other public health interventions.

This study explored state-level epidemiological risk factors for advanced-stage OCC through analysis of data from the Florida Cancer Data System (FCDS). Our goal was to examine the demographics of OCC patients, identify higher-risk geographic areas, and explore the effects of race and SES factors on advanced-stage OCC presentation to improve knowledge of specific communities at risk in Florida.

## Methods

### Data sources

This study was deemed exempt from Institutional Review Board review by the University of Miami Human Subject Research Office. We gathered de-identified SES and cancer information on patients diagnosed with OCC between 2010 and 2017 in Florida using the FCDS. The FCDS, a statewide database maintained by the Florida Department of Health and the University of Miami, reports over 130,000 cancer cases from over 6,500 facilities annually [35]. Cancer data collection including disease information, demographic data, treatment, and more is mandated at Florida hospitals and outpatient facilities. We also gathered 5-year estimates for educational attainment and income using the 2015 American Community Survey (ACS), a US Census Bureau survey that is distributed annually to randomly selected households in all 50 states, the District of Columbia, and Puerto Rico that aims to provide updated and generalizable socioeconomic data throughout the US [36].

### Inclusion/exclusion criteria

**Fig. 1:**
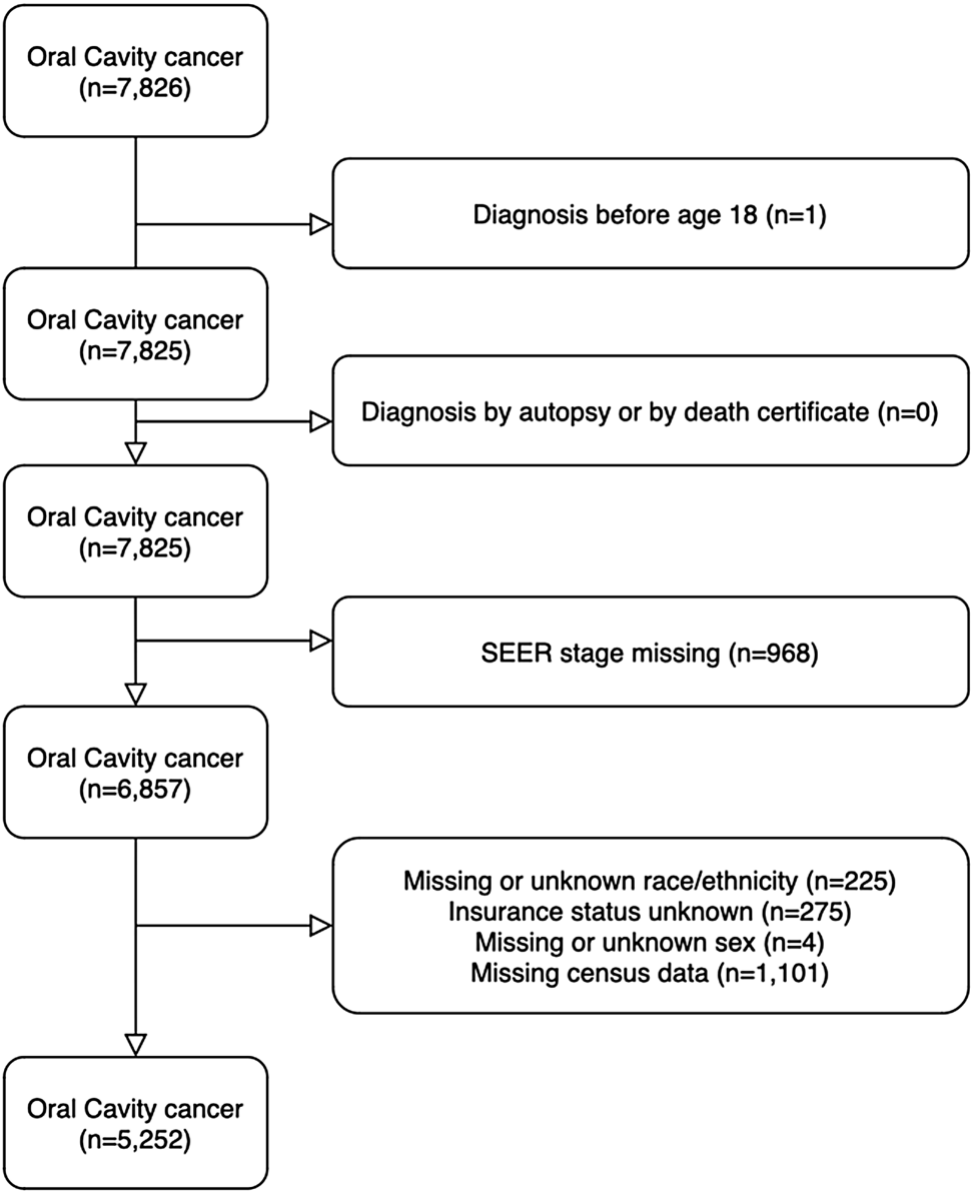
Flowchart of selection of study population of 5,252 OCC patients

Inclusion criteria was diagnosis of squamous cell OCC as indicated by *International Classification of Disease, Tenth Revision, Clinical Modification* (ICD-10 CM) primary site identifiers in the FCDS dataset. We included patients with cancer of the tongue (C02.0-C02.4, C02.8-C02.9), gum (C03.0-C03.1, C03.9), floor of mouth (C04.0-C04.1, C04.8-C04.9), palate (C05.0-C05.2, C05.8-C05.9), and other and unspecified parts of mouth (C06.0-C06.2, C06.8-C06.9). Only patients with squamous cell carcinoma (*International Classification of Diseases for Oncology, Third edition (ICD-O3)* codes 8050-8052, 8070-8076, 8078, 8082-8086) were included in analysis (Fig. 1). Exclusion criteria included patients diagnosed before 18 years of age and those diagnosed by autopsy or death certificate. Cases with missing information on SEER stage at diagnosis and/or demographic/socioeconomic variables were also excluded from the analysis.

### Variables

For this study, self-identified race and ethnic groups included non-Hispanic Whites, Hispanic Whites and Blacks. Other groups, including Native Americans and Asians, were excluded due to their small representation. Other factors included sex (male, female), age at diagnosis, marital status at time of diagnosis [never married (single, unmarried, or domestic partner), previously married (separated, divorced, widowed), married (including common law)], SEER 2010 disease stage at diagnosis [early (in-situ and local), regional, distant, unstaged], insurance status [uninsured, private, Medicaid, Medicare, TRICARE, Military/Veterans Affair (VA), Indian/Public Health Service], and tobacco cigarette smoking status (never used, current user, former user, or unknown). Florida counties were considered urban or rural based on the 2010 Census Bureau’s urban-rurality classification. While FCDS does not collect each subject’s income and education level, they collected each subject’s 2010 residential census tract, which allowed us to use ACS data as a proxy for each patient’s socioeconomic level. The following information was collected using each patient’s census tract and 2015 ACS 5-year estimates: median income (12-month) and the percentage (%) of residents with at least a bachelor’s degree. Education levels were stratified into the following quartiles: ≥ 35.91%, 23.6–35.9%, 15.21–23.6%, and ≤ 15.19%. The median annual income per census tract was stratified into the following quartiles: ≥ $59,728.1, $46,524.1–$59,728, $36,580–$46,524, and ≤ $36,579.9. Cut-off points represent the first, second, and third quartiles for the overall OCC cohort.

Our primary outcome variable was SEER stage at diagnosis, which included in-situ (confinement to basement membrane), local (no spread beyond the organ of origin), regional (direct extension to peripheral organs, tissues, and/or lymph nodes), distant (metastasis to distant organs), and unstaged disease [37]. For analysis we defined three categories: early-stage (in-situ/local), regional, and distant (Fig. 1).

### Statistical analysis

Data were analyzed using IBM SPSS Statistics, Version 28.0 (Armonk, NY). Study participants were described using counts and percentages (%), mean, and standard deviation (SD). Except for age at diagnosis, all the other independent variables were categorical variables. Pearson’s chi-square tests of independence evaluated associations of categorial variables and (1) racial/ethnic groups and (2) SEER stages at presentation (early, regional, and distant). Analysis of variance was used to compare mean of age at diagnosis among race/ethnic groups and SEER stages. Univariable and multivariable multinomial regression models were used to assess predictors of SEER stage at presentation (early (in-situ, local), regional, distant), with results reported as unadjusted (OR) and adjusted odds ratios (aOR) with 95% confidence intervals (95% CI). We tested for the multicollinearity of categorical predictor variables using linear regression. The collinearity diagnostics showed variance inflation factors between 1 and 2 on each predictor variable, indicating no significant evidence of multicollinearity (Supplementary Table 1).

In order to examine cancer burden throughout the state of Florida, geographic mapping of ratio of late and regional to early disease versus residential census tract education rates and percent uninsured was performed using the ggmap package in ArcGIS Pro 3.0 software.

## Results

FCDS query yielded 7,826 cases diagnosed between 2010 and 2017. Records were excluded due to diagnosis before 18 years of age (*n* = 1), missing SEER stage (*n* = 968), missing or unknown race/ethnicity (*n* = 225), unknown insurance status (*n* = 275), missing or unknown sex (*n* = 4), or missing census/ACS data (*n* = 1,101). Overall, 5,252 subjects were selected as our study cohort (Fig. 1).

Table 1 reports characteristics of the study cohort overall and by racial/ethnic groups. Most patients were male (61.5%), former smokers (34.0%), White non-Hispanic (82.4%), married (52.1%), publicly insured (63.3%), and urban residents (73.0%) (Table 1). The mean age of the cohort was 65.6 years (SD = 13.2) while the median income was $46,524 and median educational attainment was 23.6% of residents with at least a bachelor’s degree. In terms of disease extent at presentation, 53.1% of patients presented with early disease, 33.6% had regional disease and 13.2% had distant disease.

**Table 1:** Demographics and other characteristics of *n* = 5,252 oral cavity cancer diagnosed from 2010–2017

	All		White Non-Hispanic		White Hispanic		Black		*p*
	*n*	%	*n*	%	*n*	%	*n*	%	
All	5,252	100.0	4,328	82.4	625	11.9	299	5.7	
Age at diagnosis in years, mean (SD)	65.6 (13.2)		65.8 (13.0)		65.2 (14.3)		63.3 (12.3)		**0.01**
*Sex*									0.648
Male	3,232	61.5	2,667	61.6	376	60.2	189	63.2	
Female	2,020	38.5	1,661	38.4	249	39.8	110	36.8	
*Marital status*									** < 0.001**
Married	2,737	52.1	2,316	53.5	314	50.2	107	35.8	
Single/unmarried	1,140	21.7	878	20.3	146	23.4	116	38.8	
Separated/divorced/widowed	1,375	26.2	1,134	26.2	165	26.4	76	25.4	
*Insurance status*									** < 0.001**
Private insurance	1,583	30.1	1,308	30.2	197	31.5	78	26.1	
Uninsured	344	6.5	253	5.8	51	8.2	40	13.4	
Medicaid	381	7.3	270	6.2	71	11.4	40	13.4	
Medicare	2,748	52.3	2,331	53.9	290	46.4	127	42.5	
TRICARE	73	1.4	62	1.4	4	0.6	7	2.3	
VA/Military	110	2.1	96	2.2	9	1.4	5	1.7	
Indian/Public Health Service	13	0.2	8	0.2	3	0.5	2	0.7	
*Cigarette (tobacco) smoking status*									** < 0.001**
Never smoker	1,352	25.7	1,059	24.5	219	35.0	74	24.7	
Current smoker	1,299	24.7	1,114	25.7	101	16.2	84	28.1	
Former smoker	1,788	34.0	1,499	34.6	205	32.8	84	28.1	
Unknown	813	15.5	659	15.2	100	16.0	57	19.1	
*Median income ($)*									** < 0.001**
≥ 59,728.1	1,312	25.0	1,168	27.0	119	19.0	25	8.4	
46,524.1–59,728	1,314	25.0	1,144	26.4	131	21.0	39	13.0	
36,580.1–46,524	1,313	25.0	1,085	25.1	139	22.2	89	29.8	
≤ 36,580	1,313	25.0	931	21.5	236	37.8	146	48.8	
*Education (% with at least a bachelors)*									** < 0.001**
≥ 35.91	1,312	25.0	1,173	27.1	109	17.4	30	10.0	
23.6–35.9	1,311	25.0	1,125	26.0	143	22.9	43	14.4	
15.21–23.6	1,326	25.2	1,089	25.2	159	25.4	78	26.1	
≤ 15.2	1,303	24.8	941	21.7	214	34.2	148	49.5	
*Geographic region*									** < 0.001**
Urban	3,832	73.0	3,091	71.4	531	85.0	210	70.2	
Rural	1,420	27.0	1,237	28.6	94	15.0	89	29.8	
*SEER stage*									** < 0.001**
Early (in-situ/local)	2,791	53.1	2,375	54.9	317	50.7	99	33.1	
Regional	1,766	33.6	1,412	32.6	223	35.7	131	43.8	
Distant	695	13.2	541	12.5	85	13.6	69	23.1	

Percentages are calculated within each racial/ethnic subgroup for all variables. Bolded *p* values indicate statistical significance at *p* < 0.05

The distribution of all patient characteristics, except for sex (*p* > 0.05), was not similar among the three racial/ethnic groups. Compared to White non-Hispanics, Black patients were more likely to be younger [mean age (SD) of 63.3 (12.3) vs. 65.8 (13.0) years], uninsured (13.4% vs. 5.8%), single/unmarried (38.8% vs. 20.3%), and have regional (43.8% vs. 32.6%) or distant (23.1% vs. 12.5%) disease and they were also less likely to be former smokers (28.1% vs. 34.6%) (*p* < 0.05). White Hispanics were more likely than White non-Hispanics to be never smokers (35.0% vs. 24.5%) and less likely to be current smokers (16.2% vs. 25.7%), and more likely to be urban residents (85.0% vs. 71.4%) (*p* < 0.05). Both Black and White Hispanic patients were more likely than White non-Hispanics to be insured through Medicaid and live in a census tract with median income and education in the bottom quartile (*p* < 0.05) (Table 1).

Demographics and SES characteristics among patients diagnosed with early, regional, and distant SEER disease stage are reported in Table 2. Compared to those presenting with early disease, regional disease patients were more likely to be younger [mean age (SD) of 64.9 (13.1) vs. 66.2 (13.2) years], Black (7.4% vs. 3.5%), not married (53.9% vs. 42.2%), lower in income (27.9% vs. 21.8% in bottom quartile) and less educated (28.5% vs. 21.4% in bottom quartile) (*p* < 0.05). Compared to those presenting with early disease, patients with distant disease were more likely to be younger [mean age (SD) of 64.8 (12.9) vs. 66.2 (13.2) years], current smokers (31.5% vs. 21.5%), male (69.8% vs. 58.5%), Black (9.9% vs. 3.5%), not married (55.7% vs. 42.2%), have lower income (30.4% vs. 21.8% in bottom quartile) and lower educational attainment (29.4% vs. 21.4% in bottom quartile) (*p* < 0.05).

**Table 2:** Demographics and other characteristics of *n* = 5,252 oral cavity cancer diagnosed from 2010–2017

	All		Early		Regional		Distant		*P*
	*n*	%	*n*	%	*n*	%	*n*	%	
All	5,252	100.0	2,791	53.1	1,766	33.6	695	13.2	
Age at diagnosis in years, mean (SD)	65.6 (13.2)		66.2 (13.2)		64.9 (13.1)		64.8 (12.9)		**0.003**
*Sex*									** < 0.001**
Male	3,232	61.5	1,632	58.5	1,115	63.1	485	69.8	
Female	2,020	38.5	1,159	41.5	651	36.9	210	30.2	
*Race/ethnicity*									** < 0.001**
White Non-Hispanic	4,328	82.4	2,375	85.1	1,412	80.0	541	77.8	
White Hispanic	625	11.9	317	11.4	223	12.6	85	12.2	
Black	299	5.7	99	3.5	131	7.4	69	9.9	
*Marital status*									** < 0.001**
Married	2,737	52.1	1,614	57.8	815	46.1	308	44.3	
Single/unmarried	1,140	21.7	510	18.3	434	24.6	196	28.2	
Separated/divorced/widowed	1,375	26.2	667	23.9	517	29.3	191	27.5	
*Insurance status*									** < 0.001**
Private insurance	1,583	30.1	929	33.3	499	28.3	155	22.3	
Uninsured	344	6.5	134	4.8	134	7.6	76	10.9	
Medicaid	381	7.3	111	4.0	188	10.6	82	11.8	
Medicare	2,748	52.3	1,528	54.7	872	49.4	348	50.1	
TRICARE	73	1.4	42	1.5	26	1.5	5	0.7	
VA/Military	110	2.1	41	1.5	44	2.25	25	3.6	
Indian/Public Health Service	13	0.2	6	0.2	3	0.2	4	0.6	
*Cigarette (tobacco) smoking status*									** < 0.001**
Never smoker	1,352	25.7	767	27.5	418	23.7	167	24.0	
Current smoker	1,299	24.7	601	21.5	479	27.1	219	31.5	
Former smoker	1,788	34.0	980	35.1	589	33.4	219	31.5	
Unknown	813	15.5	443	15.9	280	15.9	90	12.9	
*Median income ($)*									** < 0.001**
≥ 59,728.1	1,312	25.0	773	27.7	386	21.9	153	22.0	
46,524.1–59,728	1,314	25.0	729	26.1	432	24.5	153	22.0	
36,580.1–46,524	1,313	25.0	680	24.4	455	25.8	178	25.6	
≤ 36,580	1,313	25.0	609	21.8	493	27.9	211	30.4	
*Education (% with at least a bachelors)*									** < 0.001**
≥ 35.91	1,312	25.0	785	28.1	388	22.0	139	20.0	
23.6–35.9	1,311	25.0	734	26.3	424	24.0	152	21.9	
15.21–23.6	1,326	25.2	675	24.2	451	25.5	200	28.8	
≤ 15.2	1,303	24.8	596	21.4	503	28.5	204	29.4	
*Geographic region*									0.229
Urban	3,832	73.0	2,054	73.6	1,289	73.0	489	70.4	
Rural	1,420	27.0	737	26.4	477	27.0	206	29.6	

Percentages are calculated within each cancer stage subgroup for all variables. Bolded *p* values indicate statistical significance at *p* < 0.05

### Regional versus early OCC

Results of UVA and MVA assessing the effects of demographic and social determinants of health variables for regional versus early-stage are displayed in Table 3 (columns A and B, respectively). Focusing on MVA results in column B, female sex (OR = 0.831, 95% CI 0.729–0.947) was protective against regional disease compared to males (*p* < 0.01). Black race (OR = 1.854, 95% CI 1.404–2.450), single/unmarried status (OR = 1.345, 95% CI 1.142–1.586), separated/divorced/widowed status (OR = 1.469, 95% CI 1.262–1.709), no insurance (OR = 1.508, 95% CI 1.150–1.978), Medicaid recipients (OR = 2.452, 95% CI 1.875–3.206), VA/military insured (OR = 1.592, 95% CI 1.016–2.494) and residence in a census tract in the bottom quarter for education (OR = 1.333, 95% CI 1.049–1.694) were independently associated with regional disease in MVA when compared to White non-Hispanics, married individuals, privately insured, and the top quartile for education, respectively (*p* < 0.05).

**Table 3:** Univariable (UVA) and Multivariable (MVA) logistic regression models assessing predictors of regional and distant disease vs. early (local/in-situ) as the reference (*n* = 5,252)

	A. UVA Regional vs. Early				B. MVA Regional vs. Early				C. UVA Distant vs. Early				D. MVA Distant vs. Early			
	OR	95% CI		*P*	OR	95% CI		*P*	OR	95% CI		*P*	OR	95% CI		*P*
Age, in years	0.993	0.988	0.998	**0.002**	0.999	0.993	1.006	0.871	0.992	0.986	0.999	**0.018**	1.002	0.993	1.011	0.623
*Race/ethnicity*																
White Non-Hispanic	1 (Ref.)				1 (Ref.)				1 (Ref.)				1 (Ref.)			
White Hispanic	1.183	0.985	1.422	0.072	1.098	0.907	1.329	0.338	1.177	0.910	1.522	0.214	1.093	0.836	1.429	0.516
Black	2.226	1.700	2.913	** < 0.001**	1.854	1.404	2.450	** < 0.001**	3.060	2.219	4.219	** < 0.001**	2.428	1.732	3.405	** < 0.001**
*Sex*																
Male	1 (Ref.)				1 (Ref.)				1 (Ref.)				1 (Ref.)			
Female	0.822	0.727	0.929	**0.002**	0.831	0.729	0.947	**0.005**	0.610	0.510	0.729	** < 0.001**	0.635	0.525	0.768	** < 0.001**
*Marital status*																
Married	1 (Ref.)				1 (Ref.)				1 (Ref.)				1 (Ref.)			
Single/unmarried	1.685	1.446	1.964	** < 0.001**	1.345	1.142	1.586	** < 0.001**	2.014	1.641	2.472	** < 0.001**	1.453	1.161	1.818	**0.001**
Separated/divorced/widowed	1.535	1.331	1.770	** < 0.001**	1.469	1.262	1.709	** < 0.001**	1.501	1.226	1.836	** < 0.001**	1.395	1.126	1.728	**0.002**
*Insurance status*																
Private insurance	1 (Ref.)				1 (Ref.)				1 (Ref.)				1 (Ref.)			
Uninsured	1.862	1.431	2.422	** < 0.001**	1.508	1.150	1.978	**0.003**	3.399	2.447	4.723	** < 0.001**	2.687	1.908	3.784	** < 0.001**
Medicaid	3.153	2.435	4.084	** < 0.001**	2.452	1.875	3.206	** < 0.001**	4.428	3.176	6.172	** < 0.001**	3.242	2.290	4.592	** < 0.001**
Medicare	1.062	0.926	1.218	0.386	1.037	0.874	1.230	0.679	1.365	1.111	1.677	**0.003**	1.342	1.044	1.725	**0.022**
TRICARE	1.152	0.698	1.902	0.579	1.063	0.639	1.768	0.815	0.714	0.278	1.832	0.714	0.632	0.244	1.636	0.344
VA/Military	1.998	1.288	3.100	**0.002**	1.592	1.016	2.494	**0.043**	3.655	2.161	6.182	** < 0.001**	2.725	1.585	4.683	** < 0.001**
Indian/Public Health Service	0.931	0.232	3.738	0.920	0.735	0.179	3.008	0.668	3.996	1.115	14.321	**0.033**	2.973	0.789	11.198	0.107
*Cigarette (tobacco) smoking status*																
Never smoker	1 (Ref.)				1 (Ref.)				1 (Ref.)				1 (Ref.)			
Current smoker	1.462	1.235	1.732	** < 0.001**	1.154	0.963	1.383	0.122	1.674	1.333	2.102	** < 0.001**	1.136	0.888	1.454	0.309
Former smoker	1.103	0.943	1.290	0.222	1.059	0.901	1.245	0.486	1.026	0.822	1.282	0.819	0.909	0.723	1.144	0.416
Unknown	1.160	0.958	1.404	0.129	1.040	0.855	1.266	0.694	0.933	0.704	1.237	0.630	0.764	0.572	1.020	0.068
*Median income (USD)*																
≥ $59,728.1	1 (Ref.)				1 (Ref.)				1 (Ref.)				1 (Ref.)			
$46,524.1–$59,728	1.187	1.001	1.407	**0.049**	1.021	0.839	1.243	0.833	1.060	0.829	1.356	0.640	0.788	0.591	1.050	0.104
$36,580–$46,524	1.340	1.130	1.589	** < 0.001**	1.024	0.829	1.273	0.828	1.323	1.041	1.680	**0.022**	0.819	0.601	1.118	0.209
≤ $36,579.9	1.621	1.367	1.922	** < 0.001**	1.047	0.823	1.333	0.709	1.750	1.386	2.211	** < 0.001**	0.886	0.631	1.245	0.487
*Education (% with at least a bachelor’s degree)*																
≥ 35.91	1 (Ref.)				1 (Ref.)				1 (Ref.)				1 (Ref.)			
23.61–35.9	1.167	0.984	1.384	0.076	1.091	0.899	1.323	0.378	1.168	0.909	1.501	0.226	1.191	0.896	1.583	0.230
15.2–23.6	1.352	1.140	1.603	** < 0.001**	1.181	0.951	1.467	0.132	1.673	1.317	2.126	** < 0.001**	1.597	1.168	2.182	**0.003**
≤ 15.19	1.707	1.441	2.024	** < 0.001**	1.333	1.049	1.694	**0.019**	1.933	1.520	2.458	** < 0.001**	1.531	1.083	2.165	**0.016**
*Geographic region*																
Urban	1 (Ref.)				1 (Ref.)				1 (Ref.)				1 (Ref.)			
Rural	1.031	0.901	1.180	0.653	1.001	0.872	1.150	0.984	1.174	0.977	1.410	0.086	1.148	0.950	1.388	0.153

OR (95% CI) = odds ratio and corresponding 95% confidence interval, P = *p* value from Wald test. Bolded *p* values indicate statistical significance at *p* < 0.05

Regarding income, UVA revealed greater odds of regional compared to early disease in the lower three income quartiles (column A, *p* < 0.05); however, these associations were not significant in MVA (column B, *p* > 0.05). Compared to those in the top educational quartile, education below the 50th percentile was associated with greater odds (*p* < 0.05) of regional vs. early disease in UVA, but this was only significant for the bottom quarter in MVA (MVA OR = 1.333, 95% CI 1.049–1.694).

Compared to never smokers, current smokers had increased odds of regional disease in UVA (OR = 1.462, 95% CI 1.235–1.732, *p* < 0.001); however, this relationship was not statistically significant in MVA (*p* > 0.05). There were no significant associations between other smoking statuses (compared to never smokers) or rural vs. urban status with regional disease (*p* > 0.05).

### Distant versus early OCC

Focusing on MVA results comparing distant to early disease (Table 3, column D), female sex was protective against distant disease (OR = 0.635, 95% CI 0.525–0.768). Black patients faced increased odds of distant disease compared to White non-Hispanics (OR = 2.428, 95% CI 1.732–3.405). Compared to married patients, those who were single/unmarried (OR = 1.453, 95% CI 1.161–1.818) or separated/divorced/widowed (OR = 1.395, 95% CI 1.126–1.728) had higher odds of distant disease. No insurance (OR = 2.687, 95% CI 1.908–3.784), Medicaid (OR = 3.242, 95% CI 2.290–4.592), Medicare (OR = 1.342, 95% CI 1.044–1.725), and VA/military coverage (OR = 2.725, 95% CI 1.585–4.683) were significantly associated with distant disease when compared to those with private insurance coverage (*p* < 0.05 for all). Similarly to regional disease, current smokers were more likely than never smokers to present with distant disease in UVA (OR = 1.674, 95% CI 1.333–2.102) (*p* < 0.001) but not in MVA. There were no significant associations with urban or rural status with distant disease.

Compared to the top income quartile, UVA revealed increased odds of distant disease vs. early disease in the bottom two income quartiles, with neither retaining significance in MVA (*p* > 0.05). Similarly to analysis comparing educational attainment in regional vs. early disease, education below the median were associated with increased odds of distant vs. early disease compared to the top quartile group (*p* < 0.05). These associations, however, persisted in MVA for both lower education groups (OR = 1.531, 95% CI 1.083–2.165 for the bottom quarter and OR = 1.597, 95% CI 1.168–2.182 for the second lowest quarter of the cohort (*p* < 0.05 for both).

### Geospatial mapping

**Fig. 2:**
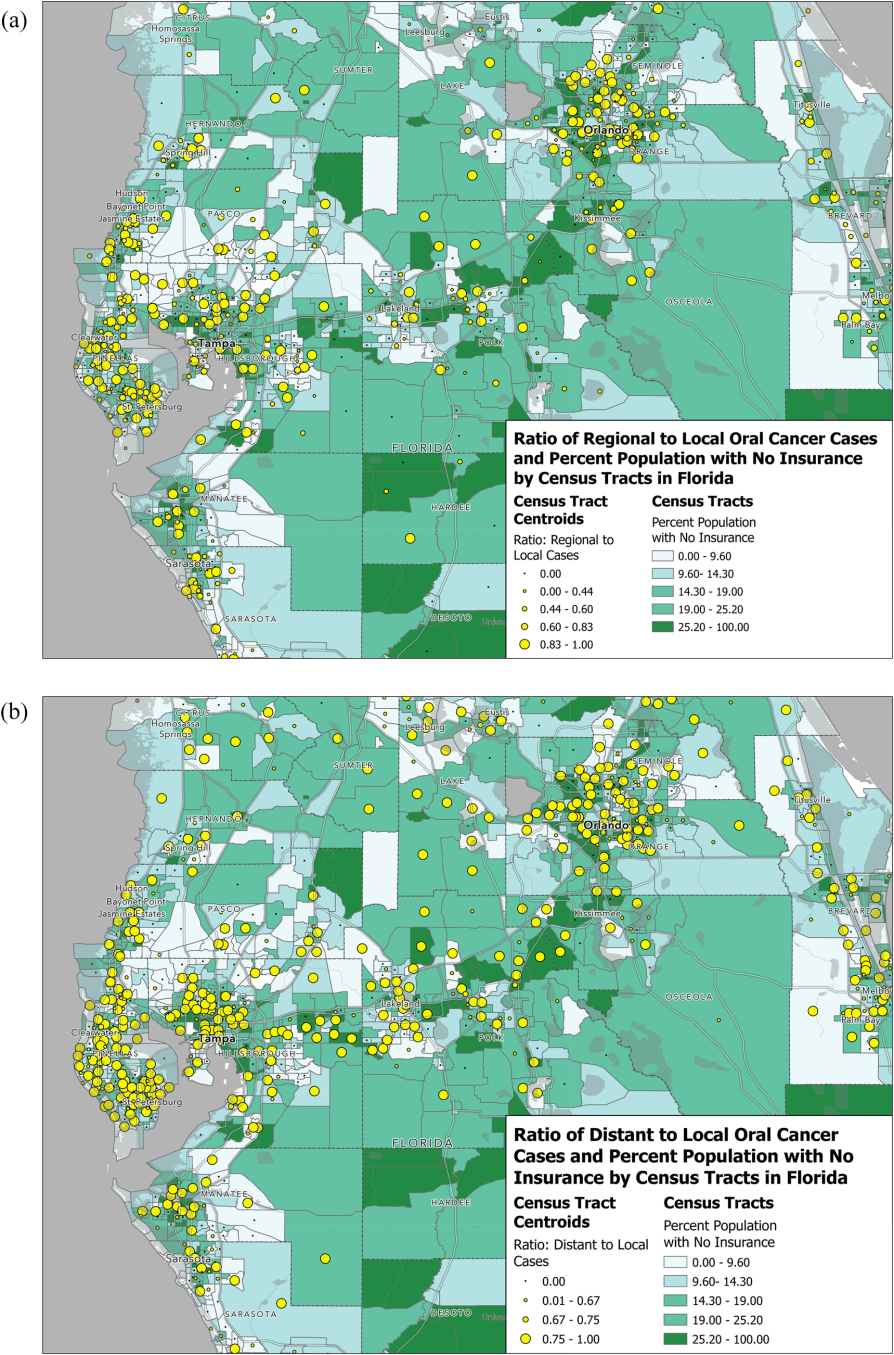
**a** Geospatial representation of the ratio of regional to early OCC diagnosis vs. uninsured percentage of census tract population. Larger circles denote a higher ratio of regional vs. early diagnosis and darker shades of green denote higher percentage of population uninsured. **b** Geospatial representation of the ratio of distant to early OCC diagnosis vs. uninsured percentage of census tract population. Larger circles denote a higher ratio of regional vs. early diagnosis and darker shades of green denote higher percentage of population uninsured

Visual representations of the ratio of regional and late stage to early diagnosis vs. percentage of uninsured individuals per census tract in Central Florida are depicted in Fig. 2a (regional vs. early) and Fig. 2b (late vs. early). Visually, larger ratios appear to be seen in areas with higher rates of uninsured individuals. Notable areas with higher ratios of regional vs. early disease can be seen in the St. Petersburg and Orlando areas (Fig. 2a). Regarding late vs. early-stage disease, a more homogenous distribution of higher ratios can be seen across population centers of Central Florida (Fig. 2b).

**Fig. 3:**
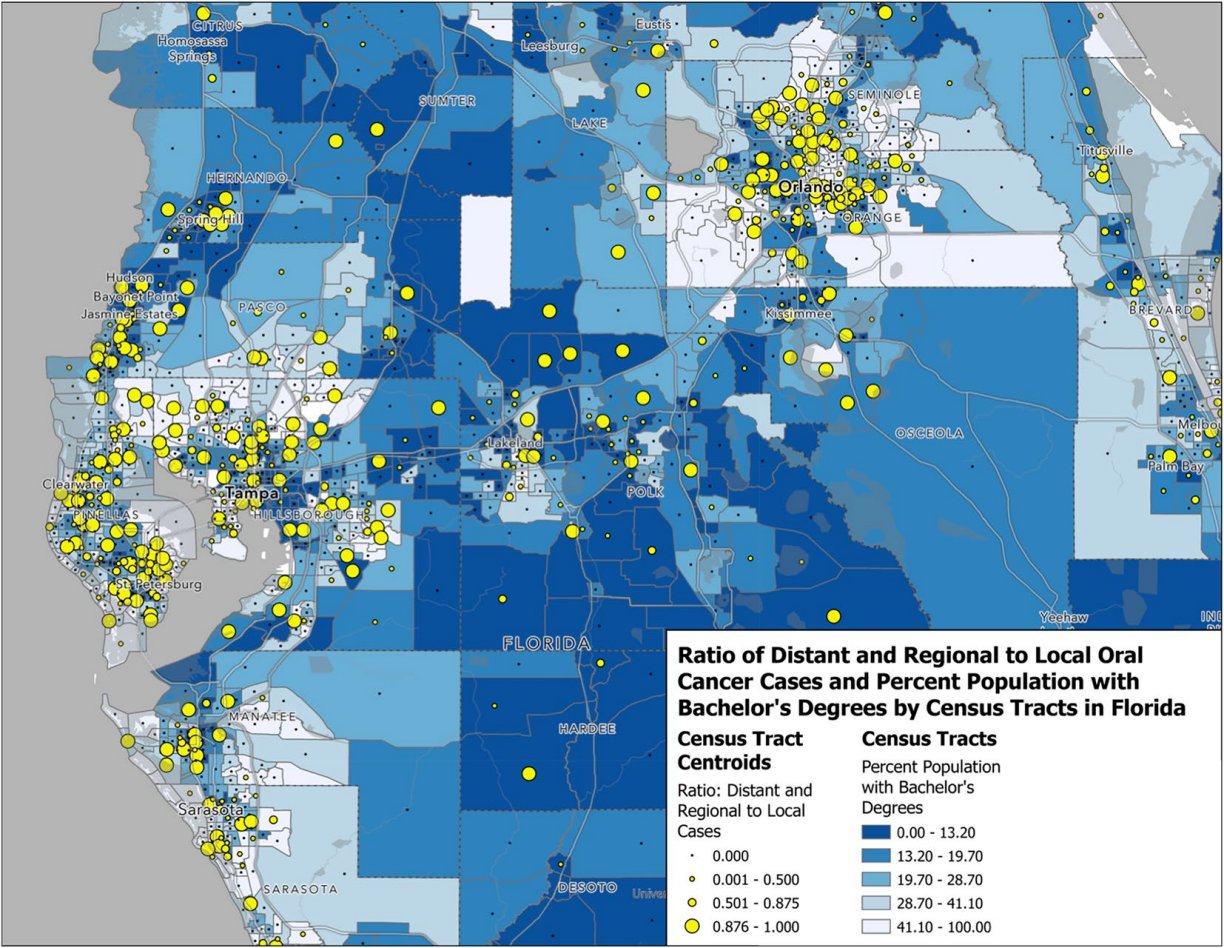
Geospatial representation of the ratio of distant and regional to early OCC diagnosis vs. percentage of census tract population with a bachelor’s degree or higher. Larger circles denote a higher ratio of distant and regional vs. early diagnosis and darker shades of blue denote lower percentage of population with a bachelor’s degree or higher

Mapping of ratios of regional and late-stage vs. early diagnosis with respect to census tract percentage with a bachelor’s degree or higher is depicted in Fig. 3. Similarly to Fig. 2a, b, higher ratios can be seen in population centers of Central Florida, with clusters of higher ratios prominent in the Orlando and Tampa Bay areas.

## Discussion

In this study, we provided a current analysis of socioeconomic factors affecting OCC incidence and stage at presentation in the state of Florida. Previously, we demonstrated disparities in laryngeal and hypopharyngeal cancer overall survival for single/unmarried individuals, while increased overall survival was observed in White Hispanics and women [38]. The present study highlights associations between Floridians’ SES disparities and OCC SEER stage at presentation.

Our study delved into the association of race and the likelihood of advanced disease. MVA indicated that Black patients (compared to White non-Hispanic patients) were more likely to present with both regional and distant disease vs. early disease. Alongside uninsured and Medicaid-insured individuals, Black patients faced the highest odds of regional and distant vs. early disease. This finding aligns with other studies conducted in the United States, demonstrating a general trend for heightened risk of advanced OCC presentation in Black Americans [12, 23, 39, 40]. Recent studies have begun to conduct comparative genomic analyses of OCC between racial groups to better understand the etiology of this heightened risk. Mezghani et al. found that patients of African ancestry had unique gene expression and DNA methylation profiles that enhance tumor aggressiveness and stated that these “differences are likely not intrinsic to race *per* se but instead are a reflection of differences in cumulative life stressors” [41]. Notably, Black patients comprised 5.7% of our study population, approximately one-third of the percentage of Black Floridians reported by the 2010 census [42]. The reason for this discrepancy is not known and is likely not related to exclusion of patients in our analysis: the number of Black patients excluded from analysis was quite small, 4.8% of excluded cases (*n* = 124) (supplementary Table 2). It is possible that Black patients have a lower overall OCC incidence (yet higher odds of advanced vs. early disease), or this may alternatively be related to differences in reporting of statistics for Black cancer patients, as has been noted in other registries [43].

Higher education and income have been explored as protective factors for OCC [21–23]. We found individuals with lower income and educational attainment to be at higher risk for regional and distant disease than those in the top income and education groups. Unlike previous studies, we found lower educational attainment, not income, to be an independent risk factor for regional and distant OCC presentation in MVA. However, the education level of the OCC population in this study was lower than that of the states’ (23.6% of our cohort had a bachelors’ degree or higher vs. 27.3% of the state’s population) according to the ACS in 2014, midway through the study period [36]. Potential explanations for these disparities may include lower levels of health literacy in less educated populations, which has been implicated in delayed diagnosis in other studies. Basharat et al. 2019 reports that many patients understand that tobacco smoke and betel nuts are carcinogenic, however, many were not aware that non-healing lesions they had may be cancerous [44, 45]. However, other studies point to decreased knowledge of risk factors as a potential contributing factor [46]. Less tangibly, differing social circles and influences among education levels may contribute to riskier behavior including smoking and lower likelihood to present for care. Individuals with lower SES may also be more likely to be uninsured or insured by Medicaid. Although income and education may be interrelated and reflect overlapping dimensions of SES, collinearity diagnostics suggest low collinearity between these variables. Accordingly, our study supports education as an independent protective factor in Florida. Current smoking was not associated with OCC stage at diagnosis after adjusting for education, other SES, and demographic variables, underscoring the importance of these factors in staging disparities. Addressing these factors independently, such as improving health literacy or accessibility to health insurance, may help form a multifaceted approach to OCC prevention directed at lower SES populations.

Unlike education and income, smoking directly causes OCC [5, 30, 47]. Although current smoking status was associated with regional and distant disease in UVA, this was not significant in MVA (compared to non-smokers, *p* > 0.05). Current smoking was associated with regional and distant disease in MVA prior to the inclusion of median income and education data, suggesting confounding and the role of smoking as a marker of low SES. This was unexpected, given the risk of OCC tied to smoking. These findings may instead reflect SES factors delaying presentation to the healthcare system. In a recent study, Cao et al. found that, although smoking has decreased in the last century in both men and women, smoking disparities have widened, with higher education linked to cessation and lower education associated with earlier smoking initiation [48]. Accordingly, as UVA results suggest, current smokers represent a population at increased risk for advanced OCC presentation in Florida. A population which, along with alcohol consumers, is less likely to report oral examination and has shown benefit from screening trials [5, 30, 31]. Further study is warranted to quantify risks associated with number of pack-years and time duration since smoking cessation as this data were unavailable through FCDS.

Both public (Medicaid and VA/military) and uninsured statuses, were associated with advanced stage at diagnosis when compared to private insurance. These results support other study findings examining insurance status with OCC and head and neck cancer outcomes [16, 18, 49]. Nationwide, uninsured and publicly insured patients face more postoperative complications and are more likely to receive treatment at hospitals with inferior outcomes [18, 49]. Uninsured individuals are less likely to have a regular source of health and dental care and are more likely to forego health care due to cost [3]. Lack of insurance is often linked to other SES risk factors such as low income and education. The interplay between veterans and oral disease is likely more complex than insurance coverage. Low SES status and smoking are unfortunately not uncommon among veterans. The link between smoking and dental concerns in this population seem to be moderated by income in other studies, suggesting a more complex interaction between smoking, income, dental disease, and perhaps delayed presentation in this population specifically [50]. However, MVA found Medicaid, VA/military coverage, and no insurance to be independent risk factors with low collinearity in our study, despite improved access to health insurance during much of the study period through the Affordable Care Act (ACA). Uninsured individuals and those with Medicaid demonstrated the highest odds of distant disease in MVA, likely due in part to the reasons listed.

Like insurance, education, and income, the influence of marital status on cancer outcomes underscores the significance of SES in cancer morbidity and mortality. Its effect as a protective factor is strong—stronger than chemotherapy in some studies [51]. The mechanisms underlying this phenomenon are likely multifaceted, potentially including factors such as reduced social support and absence of a spouse to notice visual changes tied to disease [9, 17]. Other SES inter-relationships may influence marriage status and contribute to differences between groups. For example, couples with at least a bachelor’s degree are less likely to divorce [52]. Less tangibly, some authors have suggested that college-educated couples may have warmer, more stable, and less hostile relationships [52]. These may translate to worse outcomes via connections between social support, emotional stress and tumor growth mediated by inflammatory markers and stress hormones [53]. The present study supports that married individuals have a benefit in OCC staging at diagnosis compared to other marital statuses and, after controlling for other variables, captures a unique aspect of marriage as an important indicator associated with decreased rates of advanced diagnosis. This corroborates findings of protective effects of marriage against metastatic OCC presentation in SEER studies [17]. As marriage rates have declined considerably across Florida and the entire US in recent decades, it is important for clinicians and researchers to recognize non-married status as a potential risk factor [54, 55].

We found no significant associations between advanced disease presentation and geographic location (urban versus rural) (*p* > 0.05). Although rurality was not associated with advanced disease, rural residents often experience worse OCC (and general health) outcomes [21, 23]. In Florida, rural communities face higher smoking rates, lack of education on risk factors, and less healthcare accessibility [2, 56, 57]. These disparities are also more likely to affect Black residents [39]. Consequently, there are rural areas with increased OCC burden throughout the state [58]. Similarly, studies in other regions analyzing other cancers have noted disparities in incidence and distant stage diagnosis in those of lower income and educational levels but not due to rural status [59]. Disparities may, however, be magnified in rural areas with higher levels of poverty, and less access to higher education and healthcare. These results are valuable for targeting public health interventions and research in the state, as staging differences may not be a primary driver in OCC disparities between urban and rural residents.

Geospatial mapping revealed higher rates of regional vs. early diagnosis in the Orlando and St. Petersburg areas (Fig. 2a). A visual association can be seen between percent uninsured and regional diagnosis throughout Orange, Hillsborough, Pinellas, and Manatee counties, consistent with the results of MVA. This pattern is shown more prominently in our representation of distant vs. early disease seen in Fig. 2b, correlating with relatively increased aORs seen broadly across the state. Figure 3 illustrates a more generalized association, at least in the Central Florida region, between lower levels of educational attainment and regional and distant diagnosis. It is important to note that geographic disparities may be subject to change as populations and their SES characteristics change over time. For example, insurance disparities in Orange County, where Orlando is located, has fluctuated over time. The county saw 84.8 percent of its residents insured (slightly above the state average of 83%) at the beginning of the study period, 2010. This number dropped by 2016, with only 79.7% of residents covered by any type of health insurance, significantly lower than the 2016 state average of 83.7% (*p* < 0.05) [60]. These hotspots of advanced diagnosis are likely subject to change as education rates and demographic compositions evolve over time throughout the state. Nevertheless, identifying specific geographic regions with current OCC disparities and surveilling trends in key determinants may be beneficial in planning public health interventions in areas prone to SES disparities. Percent uninsured and insured by Medicaid may be specifically useful for this purpose, as these categories were associated with some of the highest odds of regional and distant disease.

Screening trials utilizing physical examination have shown survival benefits [30, 31]. Screening programs targeted at the highest-risk individuals may be one of the most useful methods in addressing OCC staging disparities. Besides screening, effective public health efforts may include expansion of quality health care services, health literacy measures, and affordable health insurance to individuals at higher risk for advanced diseased. Specifically, health literacy initiatives regarding OCC symptom recognition and risk factors may help patients present with earlier stage cancers and further reduce their risks. Broadly, increasing access to health insurance and number of healthcare providers for patients with public health insurance and in majority Black communities may help address disparities in some of the highest-risk populations. Expanding ACA and Medicaid eligibility as well as provider compensation are specific actions that can improve accessibility to quality care, preventative services, and potentially impact staging and survival. Moreover, our geographical mapping analysis methodology could allow better targeted interventional efforts (including screening) in Florida and beyond.

One limitation of this study was the lack of data on comorbidities and patient-specific income and education available from FCDS. Median income and educational attainment were instead analyzed at the census tract level, which may differ from an individual’s income. Furthermore, quantitative information on tobacco and alcohol use was unavailable, and there is a possibility of reporting bias, particularly underreporting, related to these behaviors. Although consistent relationships between infection and OCC have not been found, data on human papillomavirus, Epstein-Barr virus, and other oncogenic viruses would be helpful for disparity analysis. Future research examining associations with non-tested variables, along with a more thorough analysis of tobacco and alcohol cessation, may enhance risk stratification. Although there are limitations, the current study provides a comprehensive analysis of OCC staging disparities in Florida. Unlike studies utilizing the SEER database, which does not report on cases from Florida, the FCDS collects information from thousands of facilities in the state, providing precise numbers not requiring extrapolation. While we had findings consistent with those in other regions, caution should be exercised if generalizing this data to states with differing populations.

## Conclusion

Our study supports SES factors as drivers of OCC staging disparities in Florida. Black race, male sex, no insurance, Medicaid coverage, non-married status, and lower education levels were associated with regional vs. early OCC disease at presentation (*p* < 0.05). These same factors, in addition to Medicare coverage, were associated with increased odds of distant cancer. While the association of smoking with OCC is established, we were surprised to learn that factors such as education and insurance status may be more indicative of advanced-stage presentation risk. This study highlights the unique profile of OCC patients in Florida, their associated risk factors, and geographic locations facing heightened OCC burden. This information will be useful in constructing screening trials, which have shown promise internationally, as well as other public health research and interventions that focus on communities with limited access to education and private insurance.

## Supplementary Information

Below is the link to the electronic supplementary material.

Supplementary file1 (DOCX 16 KB)

Supplementary file2 (DOCX 34 KB)

## Data Availability

The datasets generated during and/or analyzed during the current study are not publicly available due to patient confidentiality concerns but are available by request from FCDS (https://fcds.med.miami.edu/inc/datarequest.shtml).
