# Supplementary material for: Socioeconomic disparities: a more important risk factor for advanced-stage oral cancer in Florida than smoking?
Source: Cancer Causes Control. 2025 Mar 29;36(9):923–36. doi: 10.1007/s10552-025-01992-7 (PMC12380647; doi:10.1007/s10552-025-01992-7)
Supplement: Supplementary file 1 — Supplementary file1 (DOCX 16 KB) [file 10552_2025_1992_MOESM1_ESM.docx]

Supplementary Table 1. Collinearity diagnostics among independent variables per Table 3 calculated with education (as continuous) as the dependent variable.

| **Variable** | VIF | Tolerance |
| --- | --- | --- |
| Age | 1.388 | 0.721 |
| Race/ethnicity | 1.014 | 0.986 |
| Sex | 1.069 | 0.936 |
| Marital status | 1.128 | 0.887 |
| Insurance status | 1.268 | 0.788 |
| Cigarette (tobacco) smoking status | 1.072 | 0.932 |
| Income (census tract household median income) | 1.974 | 0.506 |
| Education (% census tract residents with bachelor's degree or higher) | 1.995 | 0.501 |
| Geographic region | 1.012 | 0.988 |

VIF: variance inflation factor; VIF= 1/(1-R_i_^2^); Tolerance= (1-R_i_^2^) where R_i_^2^ represents the unadjusted coefficient of determination for regressing the i^th^ independent variable on the remaining ones.

Multicollinearity is present when VIF is higher than 5, which implies tolerance lower than 0.2.

As reported here, the collinearity diagnostics analysis indicates that there is evidence of non-significant multicollinearity, with VIFs between 1-2 and tolerance >0.50.
