# Supplementary material for: Socioeconomic disparities: a more important risk factor for advanced-stage oral cancer in Florida than smoking?
Source: Cancer Causes Control. 2025 Mar 29;36(9):923–36. doi: 10.1007/s10552-025-01992-7 (PMC12380647; doi:10.1007/s10552-025-01992-7)
Supplement: Supplementary file 2 — Supplementary file2 (DOCX 34 KB) [file 10552_2025_1992_MOESM2_ESM.docx]

Supplementary Table 2. Characteristics of N=5,252 OCC cases included in analysis and N=2,574 OCC cases excluded from analysis due to missing data. N/a = not available

|  | **A. Cases Included in Analysis** | | **B. Excluded Cases** | | **C. Excluded Cases with Missing Stage** | | | | **D. Excluded Cases with Unstaged Disease** | |
| --- | --- | --- | --- | --- | --- | --- | --- | --- | --- | --- |
|  | n | % | n | % | n | % (of B) | | | n | % (of B) |
| All | 5,252 | 67.1 | 2,574 | 32.9 | 968 | | 37.6 | | 466 | 18.1 |
| **Age at diagnosis in years, mean (SD)** | 65.6 (13.2) | | 65.6 (12.8) | | 66.1 (12.4) | | | | 68.6 (12.1) | |
| **Sex** |  |  |  |  |  | |  | |  |  |
| Male | 3,232 | 61.5 | 1,590 | 61.8 | 595 | | 61.5 | | 272 | 58.4 |
| Female | 2,020 | 38.5 | 979 | 38.0 | 372 | | 38.4 | | 194 | 41.6 |
| Unknown/missing | 0 | 0 | 5 | 0.2 | 1 | | 0.1 | | 0 | 0 |
| **Race** |  |  |  |  |  | |  | |  |  |
| White Non-Hispanic | 4,328 | 82.4 | 1,924 | 74.7 | 793 | | 81.9 | | 373 | 80.0 |
| White Hispanic | 625 | 11.9 | 272 | 10.6 | 97 | | 10.0 | | 54 | 11.6 |
| Black | 299 | 5.7 | 124 | 4.8 | 49 | | 5.1 | | 19 | 4.1 |
| Unknown/missing | 0 | 0 | 254 | 9.9 | 29 | | 3.0 | | 20 | 4.3 |
| **Marital status** |  |  |  |  |  | |  | |  |  |
| Married | 2,737 | 52.1 | 1,168 | 45.4 | 479 | | 49.5 | | 217 | 46.6 |
| Single/unmarried | 1,140 | 21.7 | 491 | 19.1 | 210 | | 21.7 | | 95 | 20.4 |
| Separated/Divorced/Widowed | 1,375 | 26.2 | 521 | 20.1 | 228 | | 23.6 | | 124 | 26.6 |
| Unknown/missing | 0 | 0 | 394 | 15.3 | 51 | | 5.3 | | 30 | 6.4 |
| **Insurance Status** |  |  |  |  |  | |  | |  |  |
| Private Insurance | 1,583 | 30.1 | 604 | 23.5 | 271 | | 28.0 | | 85 | 18.2 |
| Uninsured | 344 | 6.5 | 114 | 4.4 | 50 | | 5.2 | | 18 | 3.9 |
| Medicaid | 381 | 7.3 | 145 | 5.6 | 61 | | 6.3 | | 17 | 3.6 |
| Medicare | 2,748 | 52.3 | 1,064 | 41.3 | 483 | | 49.9 | | 202 | 43.3 |
| TRICARE | 73 | 1.4 | 27 | 1.0 | 7 | | 0.7 | | 5 | 1.1 |
| VA/Military | 110 | 2.1 | 56 | 2.2 | 29 | | 3.0 | | 7 | 1.5 |
| Indian/Public Health Service | 13 | 0.2 | 9 | 0.3 | 1 | | 0.1 | | 2 | 0.4 |
| Insurance, NOS | 0 | 0 | 239 | 9.3 | 35 | | 3.6 | | 13 | 2.8 |
| Unknown/missing | 0 | 0 | 316 | 12.3 | 31 | | 3.2 | | 117 | 25.1 |
| **Cigarette (tobacco) smoking status** |  |  |  |  |  | |  | |  |  |
| Never smoker | 1,352 | 25.7 | 673 | 26.1 | 264 | | 27.3 | | 104 | 22.3 |
| Current Smoker | 1,299 | 24.7 | 589 | 22.9 | 244 | | 25.2 | | 77 | 16.5 |
| Former Smoker | 1,788 | 34.0 | 840 | 32.6 | 331 | | 34.2 | | 144 | 30.9 |
| Unknown/missing | 813 | 15.5 | 472 | 18.3 | 129 | | 13.3 | | 141 | 30.3 |
| **Median Income ($)** |  |  |  |  |  | |  | |  |  |
| ≥59,728.1 | 1,312 | 25.0 | 0 | 0 | 0 | | 0 | | 0 | 0 |
| 46,524.1-59,728 | 1,314 | 25.0 | 0 | 0 | 0 | | 0 | | 0 | 0 |
| 36,580.1-46,524 | 1,313 | 25.0 | 0 | 0 | 0 | | 0 | | 0 | 0 |
| ≤36,580 | 1,313 | 25.0 | 0 | 0 | 0 | | 0 | | 0 | 0 |
| Unknown/missing | 0 | 0 | 2,574 | 100.0 | 968 | | 100.0 | | 466 | 100.0 |
| **Education (% with at least a bachelors)** |  | |  |  |  | |  | |  |  |
| ≥35.91 | 1,312 | 25.0 | 1 | 0 | 0 | | 0 | | 0 | 0 |
| 23.6-35.9 | 1,311 | 25.0 | 0 | 0 | 0 | | 0 | | 0 | 0 |
| 15.21-23.6 | 1,326 | 25.2 | 0 | 0 | 0 | | 0 | | 0 | 0 |
| ≤15.2 | 1,303 | 24.8 | 1 | 0 | 0 | | 0 | | 0 | 0 |
| Unknown/missing | 0 | 0 | 2,574 | 99.9 | 968 | | 100.0 | | 466 | 100.0 |
| **Geographic Region** |  |  |  |  |  | |  | |  |  |
| Urban | 3,832 | 73.0 | 1,850 | 71.9 | 694 | | 71.7 | | 355 | 76.2 |
| Rural | 1,420 | 27.0 | 724 | 28.1 | 274 | | 28.3 | | 111 | 23.8 |
| Unknown/missing | 0 | 0 | 0 | 0 | 0 | | 0 | | 0 | 0 |
| **SEER stage** |  |  |  |  |  | | |  |  |  |
| Early (in-situ/local) | 2,791 | 53.1 | 580 | 22.5 | n/a | | | n/a | n/a | n/a |
| Regional | 1,766 | 33.6 | 408 | 15.9 | n/a | | | n/a | n/a | n/a |
| Distant | 695 | 13.2 | 152 | 5.9 | n/a | | | n/a | n/a | n/a |
| Unstaged | 0 | 0 | 466 | 18.1 | n/a | | | n/a | 466 | 100.0 |
| Missing stage | 0 | 0 | 968 | 37.6 | 968 | | | 100.0 | n/a | n/a |
